# Exact *vs.* Approximate Computation: Reconciling Different Estimates of *Mycobacterium tuberculosis* Epidemiological Parameters

**DOI:** 10.1534/genetics.113.158808

**Published:** 2014-02-04

**Authors:** R. Zachariah Aandahl, Tanja Stadler, Scott A. Sisson, Mark M. Tanaka

**Affiliations:** * School of Mathematics and Statistics, University of New South Wales, Sydney, NSW 2052 Australia; † School of Biotechnology and Biomolecular Sciences and Evolution & Ecology Research Centre, University of New South Wales, Sydney, NSW 2052 Australia; ‡ Department of Biosystems Science and Engineering, Eidgenössische Technische Hochschule, Zürich, 4058 Basel, Switzerland

**Keywords:** approximate Bayesian computation, *Mycobacterium tuberculosis*, reproductive number, summary statistics, IS*6110*

## Abstract

Exact computational methods for inference in population genetics are intuitively preferable to approximate analyses. We reconcile two starkly different estimates of the reproductive number of tuberculosis from previous studies that used the same genotyping data and underlying model. This demonstrates the value of approximate analyses in validating exact methods.

TWO previous methods for analyzing *Mycobacterium tuberculosis* infection and evolution produced conflicting estimates of the effective reproductive number, *R*. Tanaka *et al.* (2006) used approximate Bayesian computation (ABC) (Beaumont 2010; Csilléry *et al.* 2010) with two summary statistics to estimate this parameter using data from San Francisco (Small *et al.* 1994), yielding *R* = 3.4 (95% C.I. 1.4, 79.7). Stadler (2011) derived an exact likelihood to analyze the same data within a Bayesian framework, giving the estimate *R* = 1.02 (95% C.I. 1.01,1.04). If this discrepancy is due to the approximation in ABC methods, it would call into question the reliability of ABC in other studies using similar summary statistics and models. We therefore investigate and resolve this discrepancy here.

In both methods, the underlying process is a continuous time birth–death process with mutations occurring (at rate *θ* per infection per year) according to the assumption of infinite alleles. A birth event corresponds to a transmission event (with rate *λ* per infection per year) of tuberculosis while a death event represents death or recovery (with rate *μ* per infection per year). Under the method of Tanaka *et al.* (2006) (henceforth “ABC06 method”), inference is performed using ABC and implemented with Markov chain Monte Carlo (MCMC) (Marjoram *et al.* 2003; Sisson and Fan 2011). The process is simulated from a single infectious individual until either extinction occurs or the infectious population reaches a size *N*, at which point a sample of size *n* is taken. Two summary statistics are computed: the number of distinct genotypes in the sample and the virtual heterozygosity or gene diversity. A distance between observed and simulated statistics is computed to assess whether a parameter set should be accepted, leading to an approximate posterior parameter distribution.

The method of Stadler (2011) (henceforth “Tree11 method”) derives an expression for the likelihood of a transmission tree with associated mutations, giving rise to a sample of genotypes (Equation 3 in Stadler (2011)). It is assumed that the epidemic started at a random time *t*_0_ in the past and each presently infected individual is included into the genotype sample with probability *ρ* = *n*/*N*. MCMC is used to explore the space of parameters and obtain a Bayesian posterior parameter distribution. We highlight here that the ABC06 and Tree11 method rely on the same model, up to the length of the epidemic and the exact sampling procedure. The ABC06 method assumes the epidemic spreads until *N* individuals are infected and then *n* isolates are taken. The Tree11 method assumes that the epidemic starts at a random time in the past, and an isolate is sampled from an individual with probability *ρ*.

Stadler (2011) proposed that the discrepancy between the methods was due to a loss of information from the data when using nonsufficient summary statistics in ABC06. Here, we assess the choice of summary statistics in Tanaka *et al.* (2006) by comparing the ABC method against the exact likelihood of observing data sampled from a population using the same simulation process as the ABC method. We call this the “Exact method.” Following Stadler (2011) who showed that the mutation rate, *θ*, cannot be estimated from snapshot genotyping data, we fix *θ* = 0.198. We also found that ABC06 with uninformative priors for the correlated parameters *λ* and *μ* consistently leads to similar estimates of *R*, regardless of the parameters used to simulate the data. We were able to rectify this problem either by setting *μ* to a constant or by using an informed prior (we call this form the “ABC method”). Here, we fix *μ* = 0.52 as the sum of estimates of the rates of self cure, death from causes other than tuberculosis, and death from untreated tuberculosis (Cohen and Murray 2004; Luciani *et al.* 2009).

The Exact method is as follows. Define the observed data G_*0*_ as a sample of isolates of size *n*, *c* as the number of distinct genotypes in G_*0*_, and *n_i_* as the number of instances of genotype *i* in G_*0*_ so that $n=\sum_{i=1}^{c}n_{i}$. Let G_s_ be the unobserved population of size *N*, *G* the number of distinct genotypes in G_s_, and *X_i_* the number of instances of genotype *i* in G_s_ so that $N=\sum_{i=1}^{G}X_{i}$. The posterior distribution of the effective reproductive number, *R* = *λ*/*μ*, given G_*0*_, is $\pi \left(R|G_{\text{0}}\right)=\int \pi \left(R,G_{\text{s}}|G_{\text{0}}\right)dG_{\text{s}}\propto \int \pi \left(G_{0}|R,G_{\text{s}}\right)\pi \left(R,G_{\text{s}}\right)dG_{\text{s}}\propto \int \pi \left(G_{\text{0}}|G_{\text{s}}\right)\pi \left(G_{\text{s}}|R\right)\pi \left(R\right)dG_{\text{s}}.$ Conditional on *G* ≥ *c*, we define the set P as all of the *c* sized subsets in {1, 2,…, *G*} and *p*(*i*) as the *i*th value of subset *p* in P. The probability that G_0_ came from G_s_ is

$$\pi \left(G_{0}|G_{\text{s}}\right)=\sum_{p\in P}\left(\frac{\prod_{i=1}^{c}\left(\begin{array}{c} X_{p\left(i\right)}\\ n_{i} \end{array}\right)}{\left(\begin{array}{c} N\\ n \end{array}\right)}\right).$$ (1)

We used Equation 1 to sample from *π*(*R*, G_s_ | G_*0*_) and estimate *π*(*R* | G_*0*_) for each of 100 simulated data sets generated from a known value of *R* and used standard MCMC methods. We compared the resulting posterior distributions to those obtained using the ABC and Tree11 methods via a two-sample Kolmogorov–Smirnov test, based on posterior samples of size 100. Box plots of the resulting *P*-values (Figure 1A) indicate that the posteriors from the ABC method are similar to those from the Exact approach, while the posteriors from the Tree11 method are clearly different in each case. More precisely, we found that posteriors estimated using the ABC method were centered on the true, known values of *R*, but those estimated using the Tree11 method were shifted to the left (*e.g.*, Figure 1, B–E). We identified two problems that affect inference when using the model from Stadler (2011).

**Figure 1:**
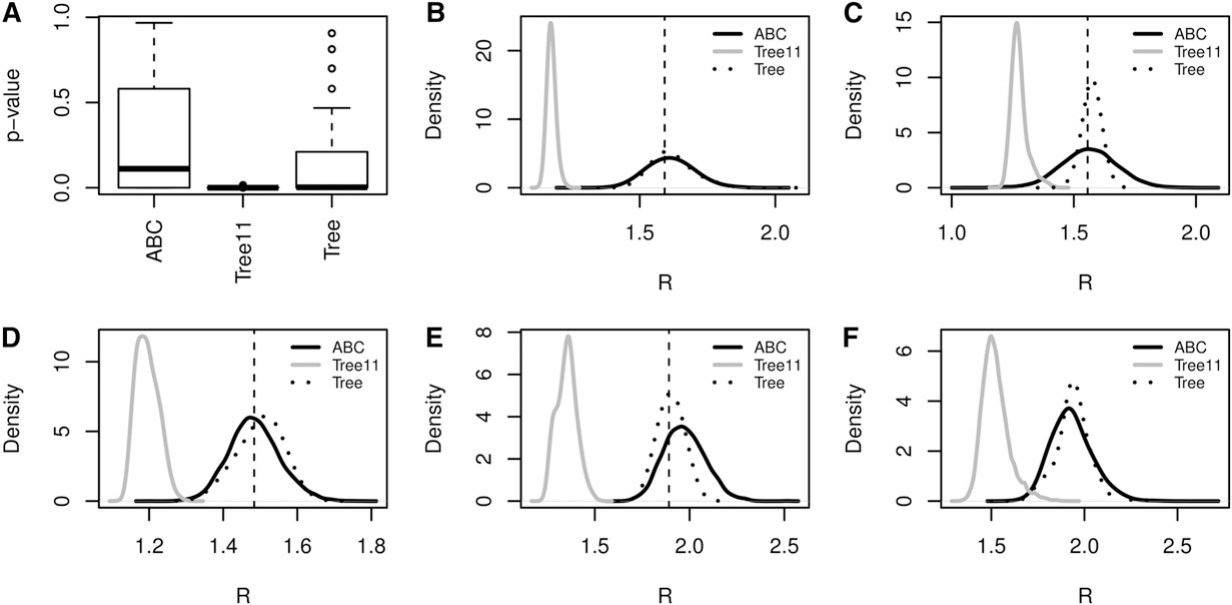
Estimation of the effective reproductive number *R* using the (corrected) ABC, Exact, Tree11, and Tree methods. In all analyses, *θ* = 0.198. (A) Boxplots of 100 replicates of *P*-values from two-sample Kolmogorov–Smirnov tests, comparing the posterior distribution of *R* under the ABC, Tree11, and Tree methods with the Exact method. Each replicate *P*-value was based on data generated with *R* = 4, *μ* = 0.52, and *ρ* = 0.1. (B) Estimates of the posterior distribution of *R* using the ABC, Tree11, and Tree methods, based on simulated data with *R* = 1.60 (indicated by the vertical dashed line), *μ* = 0.52, and *ρ* = 0.05. (C) As for B, but using *R* = 1.55, *μ* = 0.34. (D) As for B, but using *R* = 1.48, *μ* = 0.62. (E) As for B, but using *R* = 1.89, *μ* = 0.75. (F) As for B, but using data from Small *et al.* (1994), and with the prior *μ* ∼ *N*(0.52, 0.004167).

First, *f*(T|*t*_0_) (*cf*. Stadler (2011) p. 666) gives the probability of an oriented tree, while the sampler operates on vectors of branching times, T_v_ (one vector per genotype). To correct this we derived the distribution of the vectors T_v_|*t*_0_. We calculated the probability of a labeled tree $\left(f\left(T|t_{0}\right)\left(2^{\left(n-1\right)}/n!\right)\right)$, summed over all within-genotype labeled trees $\left(\prod_{i=1}^{c}\left[n_{i}!\left(n_{i}-1\right)!/2^{n_{i}-1}\right]\right)$, and summed over the number of ways (*m_i_*) in which a genotype cluster (*i*) may connect to a tree $\left(\prod_{i=1}^{c-1}m_{i}\right)$ to obtain

$$f\left(T_{\text{v}}|t_{0}\right)=f\left(T|t_{0}\right)\frac{2^{n-1}}{n!}\prod_{i=1}^{c}\frac{n_{i}!\left(n_{i}-1\right)!}{2^{n_{i}-1}}\prod_{i=1}^{c-1}m_{i}\propto f\left(T|t_{0}\right)\prod_{i=1}^{c-1}m_{i}.$$ (2)

Second, we found that the state of the MCMC sampler would become trapped in local maxima due to an inefficient proposal distribution. To address this, we modified the proposal to uniformly resample the genotype cluster vectors of branching times at each stage of the algorithm. We refer to this adjusted form of the Tree11 approach as the Tree method.

We tested the accuracy of the ABC, Tree11, and Tree methods by computing the posterior distribution for *R* based on data generated from TreeSim (Stadler 2010) with an infinite alleles model of mutation. We then calculated the mean squared error (MSE) of the resulting posteriors compared to the true value of *R*. Table 1 presents the mean MSE and standard errors for each method based on 10 replicate data sets. An example of the posterior distributions resulting from one of the replicated data sets is shown in Figure 1B. Additional posterior distributions using different parameter combinations are shown in Figure 1, C–E. Very clearly, the ABC and Tree methods perform similarly well, and both outperform the Tree11 method (see also Figure 1A).

**Table 1:** Average mean squared error (MSE) estimates of the posterior distribution of *R*, based on 10 replicate analyses, using data simulated with *θ* = 0.198, *N* = 5000

	Mean MSE	SE of mean MSE
ABC	14.6 × 10^−3^	3.0 × 10^−3^
Tree11	86.9 × 10^−3^	39.3 × 10^−3^
Tree	13.9 × 10^−3^	4.4 × 10^−3^

The parameter *μ* for each of the 10 tests was chosen uniformly between 0.3 and 8, and *R* was chosen uniformly between 1 and 2.

Finally, we reanalyzed the observed data taken from the IS*6110* isolates in San Francisco in Small *et al.* (1994), but by fixing the value of mutation rate *θ* = 0.198 and using the Gaussian prior *μ* ∼ *N*(0.52, *σ*^2^ = 0.0125/3) for the death/recovery rate. The prior standard deviation corresponds to the standard deviation of the triangular distribution used in Dye and Espinal (2001). Figure 1F shows the resulting posterior distributions of *R* using the ABC, Tree11, and Tree methods. The original Tanaka *et al.* (2006) estimate using the unmodified ABC method, trying to estimate all parameters, is *R* = 3.4 (95% C.I. 1.4, 79.7). The estimate from the model from Stadler (2011) is *R* = 1.63 (95% C.I. 1.32, 1.94). However, using the corrected methods, the estimate using the ABC method is *R* = 2.10 (95% C.I. 1.54, 2.66), and the estimate using the Tree method is *R* = 2.05 (95% C.I. 1.55, 2.53). The point estimates and credible intervals from the posteriors from the ABC and the Tree method are in close agreement.

We have shown that the ABC analysis of Tanaka *et al.* (2006) based on the method of Marjoram *et al.* (2003) is valid as long as an informative prior is used for two of the parameters (here, the mutation rate *θ* and the death and recovery rate *μ*). The modified priors eliminate potential problems in the ABC and Tree approaches due to the strong correlation between *μ* and *λ*. This correction addresses the concern raised by Stadler (2011); that is, there is no substantial loss of information through the choice of summary statistics in the ABC method. Finally, we have improved the method of Stadler (2011) by modifying the mechanism of proposing new trees within the MCMC sampler to prevent it from converging to local maxima. In combination, these adjustments have reconciled the discrepancies between Tanaka *et al.* (2006) and Stadler (2011); the methods now perform equivalently.

Exact likelihood methods such as that of Stadler (2011) are generally preferable to ABC, which is an approximate inferential procedure. Here, however, we have demonstrated the value of using approximate methods to validate exact computational methods based on models with high-dimensional latent variables. For this setting, the ABC method has similar accuracy to and better computational efficiency than the Tree method. A further advantage of the ABC method is that it can easily be extended to more complex models. Recent work generalizing the coalescent to incorporate SIR dynamics (Volz *et al.* 2009; Rasmussen *et al.* 2011) presents a promising alternative approach for estimating parameters from genetic data under more realistic epidemiological models. Comparison of the coalescent SIS approach to fully stochastic models has been addressed elsewhere (Leventhal *et al.* 2014) and would be an important issue to explore further in the future.
